# Potential Modulation of Polygoni Cuspidati Rhizoma et Radix on Breast Cancer Resistance Protein and Marked Alteration on Methotrexate Pharmacokinetics

**DOI:** 10.3390/ph18111636

**Published:** 2025-10-29

**Authors:** Yu-Chi Hou, Pei-Ying Li, Shiuan-Pey Lin, Pei-Wen Hsu, Meng-Hao Wu, Chung-Ping Yu

**Affiliations:** 1 School of Pharmacy, College of Pharmacy, China Medical University, Taichung 406040, Taiwan; houyc@mail.cmu.edu.tw (Y.-C.H.); anag8066@gmail.com (P.-Y.L.); splin@mail.cmu.edu.tw (S.-P.L.);; 2 Department of Pharmacy, China Medical University Hospital, Taichung 404327, Taiwan; 3 Institute of Chinese Pharmaceutical Sciences, China Medical University, Taichung 404333, Taiwan

**Keywords:** breast cancer resistance protein, herb-methotrexate interaction, pharmacokinetics, polygoni cuspidati rhizoma et radix

## Abstract

**Background/Objectives**: Polygoni Cuspidati Rhizoma et Radix (PCRR) is an herb and a source of a resveratrol-containing dietary supplement. Breast cancer resistance protein (BCRP) is an ATP-binding cassette transporter involved in numerous drug-related pharmacokinetic interactions. This study used both in vivo and *in vitro* models to investigate the modulation effect of PCRR ingestion on BCRP. **Methods**: Three groups of rats were orally administered methotrexate (MTX), a probe substrate of BCRP, without and with PCRR at 1.0 g/kg and 2.0 g/kg in a parallel design, and the MTX pharmacokinetics were compared among three treatments. The modulation effects of PCRR and its serum metabolites (PCRRM) on BCRP were assayed by *in vitro* models. **Results**: PCRR at 1.0 g/kg and 2.0 g/kg significantly decreased the area under the serum level–time curve from 0 to 240 min (AUC_0-240_) of MTX by 31% and 58%, respectively; 2.0 g/kg of PCRR markedly increased the area under the serum level–time curve from 240 to 2880 min (AUC_240-2880_) and the mean residence time (MRT) of MTX by 39% and 74%, respectively. The results of *in vitro* assays indicated that PCRR enhanced the function of BCRP by 33~48%; on the contrary, PCRRM reduced the function of BCRP by 200~209%. **Conclusions**: PCRR activated BCRP, whereas PCRRM inhibited BCRP, thereby the coadministration of PCRR reduced both the absorption and excretion of MTX in rats. In clinical practice, the concurrent use of PCRR with critical BCRP substrate drugs should be avoided.

## 1. Introduction

Polygoni Cuspidati Rhizoma et Radix (PCRR, the dried rhizomes and roots of *Polygonum cuspidatum* Sieb. et Zucc.) is an herb and has been a source of a resveratrol-containing dietary supplement in recent decades [1,2]. Clinically, PCRR is often prescribed for the treatment of various inflammatory diseases, hepatitis and tumors in East Asia and North America [3,4,5]. The daily dose of PCRR is 9–20 g [6]. The major constituents of PCRR are resveratrol, emodin and their glycosides [7], which exhibit various beneficial bioactivities, including anti-inflammation, anti-virus, anti-bacteria, anti-cancer and neuroprotection [3,8].

Breast cancer resistance protein (BCRP) is known as one of the key members of ATP-binding cassette (ABC) transporters, present in the intestine, liver and kidney. BCRP transports a broad specificity of drugs and is involved in the pharmacokinetics of numerous medicines and drug interactions [9,10]. Methotrexate (MTX) is a substrate of BCRP [11] and one of the WHO Model List of Essential Medicines often prescribed for the treatment of autoimmune and neoplastic diseases, but with a narrow therapeutic window [12]. During treatment with MTX, life-threatening hepatotoxicity, pulmonary toxicity, myelosuppression and nephrotoxicity may occur [13,14,15]. Therefore, any modulation of BCRP might exert significant impacts on the pharmacokinetics and clinical outcome of MTX.

Nowadays, several polyphenol-rich herbs (e.g., *Magnolia officinalis* and *Scutellaria baicalensis*) and their polyphenolic constituents (e.g., magnolol, honokiol, baicalin and resveratrol) have shown potential modulations on BCRP and led to significant influences on the pharmacokinetics of BCRP substrate drugs [16,17,18,19,20,21,22]. Furthermore, the conjugated metabolites of polyphenols were verified as substrates and/or modulators of BCRP, such as resveratrol glucuronides [16,23].

In our rat study, MTX was selected as an in vivo probe substrate of BCRP. Based on previous *in vitro* findings that resveratrol and resveratrol glucuronides modulated BCRP [16,24], we hypothesized that PCRR and its serum metabolites (PCRRM) might modulate BCRP; the coadministration of PCRR likely led to significant alteration on the pharmacokinetics of MTX. Therefore, this study investigated the influence of PCRR coadministration on the pharmacokinetics of MTX in rats, and the modulation effects of PCRR and PCRRM on BCRP were investigated by using *in vitro* assays.

## 2. Results

### 2.1. Quantitation of Resveratrol and Emodin in PCRR Decoction

**Figure 1:**
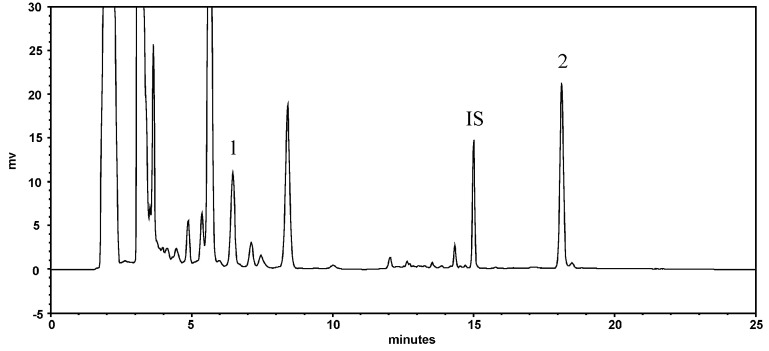
HPLC chromatogram of PCRR decoction: 1: resveratrol; 2: emodin; IS: butyl paraben (internal standard).

Figure 1 shows the HPLC chromatogram of resveratrol and emodin in PCRR decoction. The quantitation results showed that the concentration of resveratrol and emodin in PCRR were 276.1 and 564.5 μg/g, respectively.

### 2.2. PCRR-MTX Pharmacokinetic Interaction Study in Rats

**Figure 2:**
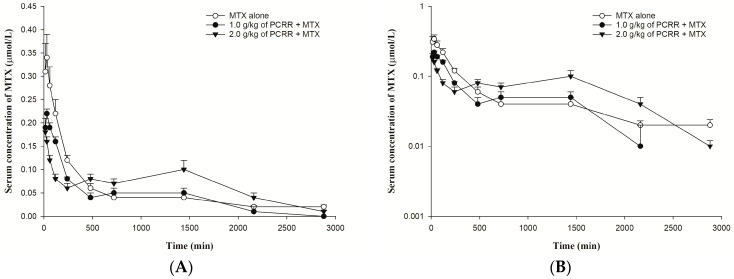
(**A**): Mean (±S.E.) serum level–time profiles of MTX (5.0 mg/kg) after dosing MTX alone (○, *n* = 6) and coadministrations with 1.0 g/kg (●, *n* = 6) and 2.0 g/kg (▼, *n* = 5) of PCRR and (**B**): the semi-log diagram of (**A**).

The serum profiles of MTX and its semi-log diagram of rats orally given MTX with and without PCRR are shown in Figure 2A,B, respectively. The pharmacokinetic parameters of MTX after three treatments are listed in Table 1. The results indicated that 1.0 g/kg of PCRR markedly reduced the AUC_0-240_ of MTX by 31%. In contrast, 2.0 g/kg of PCRR markedly reduced the C_max_ and AUC_0-240_ of MTX by 46% and 58%, whereas the AUC_240-2880_ and MRT markedly increased by 39% and 74%, respectively.

### 2.3. Function Assay of BCRP After Treatments with PCRR and PCRRM

**Figure 3:**
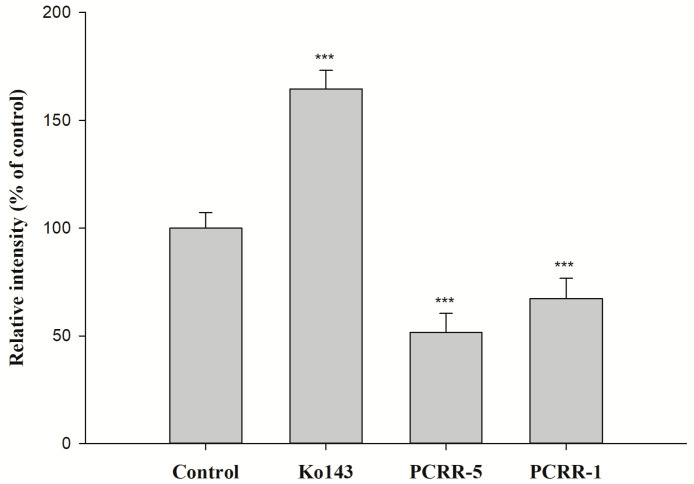
The effect of PCRR (mg/mL) on the intracellular accumulation of MXR in MDCKII-BCRP cells. Ko143: a BCRP inhibitor and MXR: a BCRP fluorescent substrate. *** *p* < 0.001; relative intensity (% of control): percentage of the fluorescence in the treatment group compared to that in control group. Data expressed as mean ± S.D. of three determinations.

**Figure 4:**
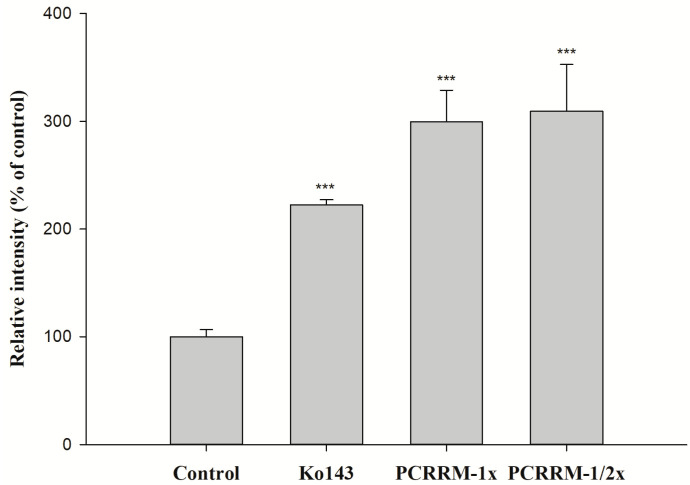
The effect of PCRRM (1- and 1/2-fold serum level) on the intracellular accumulation of MXR in MDCKII-BCRP cells. Ko143: a BCRP inhibitor and MXR: a BCRP fluorescent substrate. *** *p* < 0.001; relative intensity (% of control): percentage of the fluorescence in the treatment group compared to that in control group. Data expressed as mean ± S.D. of three determinations.

The effects of PCRR and PCRRM on the function of BCRP are shown in Figure 3 and Figure 4, respectively. The results indicated that PCRR at 5.0 and 1.0 mg/mL markedly reduced the intracellular accumulation of MXR by 48% and 33%, respectively, and Ko143, a positive BCRP inhibitor, increased that by 65%. On the contrary, PCRRM at 1- and 1/2-fold serum levels markedly increased the intracellular accumulation of MXR by 200% and 209%, respectively, and Ko143 increased that by 122%.

## 3. Discussion

To our knowledge, this is the first study investigating the modulation effects of both PCRR and its serum metabolites (PCRRM) on BCRP. In this study, MTX was orally administered to rats serving as a probe substrate of BCRP for assessing the probable in vivo modulation on BCRP after PCRR ingestion. A comparison of the pharmacokinetic data of MTX among three groups showed that both doses of PCRR (2.0 g/kg and 1.0 g/kg) significantly decreased the early exposure (AUC_0-240_) of MTX by 58% and 31%, indicating that the absorption of MTX was apparently reduced by PCRR. Interestingly, 2.0 g/kg of PCRR significantly increased the late exposure (AUC_240-2880_) and MRT of MTX by 39% and 74%, respectively, whereas 1.0 g/kg of PCRR did not lead to any significant differences in the AUC_240-2880_ and MRT, clearly indicating that the elimination of MTX was markedly inhibited by 2.0 g/kg of PCRR, but not obvious with 1.0 g/kg of PCRR.

Observation of the three serum profiles of MTX following different treatments indicated that the absorption profiles of two treatment groups with PCRR were apparently lower than that of the control, wherein the profile of 2.0 g/kg of PCRR was the lowest one, indicating that the absorption of MTX was reduced dose-dependently by PCRR. In regard to the later elimination phase of MTX, the profile of coadministration with 2.0 g/kg of PCRR became much higher than that of the control at around 12 h after dosing of MTX, implying that this dose of PCRR markedly inhibited the elimination of MTX, which had overwhelmed its earlier effect of reducing the absorption of MTX. As for the dose of PCRR at 1.0 g/kg, the absorption profile of MTX was between the other two treatment groups, but the elimination profile was almost superposable with that of the control group, which could be accounted for by the fact that the inhibition effect on the elimination of MTX was canceled out by the reduced absorption. Therefore, we conclude that coadministration of PCRR inhibited both the absorption and elimination of MTX in rats whether PCRR was coadministered at 2.0 g/kg or 1.0 g/kg.

The function assay of BCRP indicated that PCRR at 5.0 mg/mL and 1.0 mg/mL significantly decreased the intracellular accumulation of MXR by 48% and 33%, indicating that PCRR activated BCRP-mediated efflux transport. Thus, we could speculate that the reduced absorption of MTX by PCRR might be in part accounted for by the activation of BCRP in the intestine. After PCRR was absorbed, the major molecules in the circulation were mainly the conjugated metabolites of resveratrol and emodin, whose physicochemical properties were quite different from their parent molecules in the PCRR decoction. Therefore, this study used the characterized PCRRM containing the conjugated metabolites of resveratrol (6.3 nmol/mL) and emodin (3.6 nmol/mL) (HPLC chromatograms of PCRRM in rat serum after PCRR ingestion are presented in Figure S1 of the Supplementary Material) [25] to mimick the virtual molecules in contact with the kidney where 80–90% of MTX was excreted [26]. The results of this *ex vivo* study showed that PCRRM at 1- and 1/2-fold serum levels significantly increased the intracellular accumulation of MXR by 200% and 209%, respectively, indicating that PCRRM markedly inhibited BCRP-mediated efflux transport, which was opposite to the activation effect of PCRR. We speculated that the conjugated metabolites of resveratrol and emodin in PCRRM were the major inhibitors of BCRP [23]. Consistently, a previous study had indicated that resveratrol metabolites inhibited the function of BCRP and increased the bioavailability of warfarin, a BCRP substrate [20]. Therefore, we conclude that the marked inhibition effect of PCRRM on BCRP could in part explain the enhanced late exposure of MTX, which was attributed to the hampered excretion of MTX via BCRP in the kidney.

Concerning the precise molecular mechanism of the modulation of BCRP, this study has a limitation of lacking evaluations on the direct binding site competition, allosteric modulation and gene expression changes in BCRP, which remained unknown and are necessary to be investigated for predicting the unknown probable pharmacokinetic interactions of PCRR with BCRP substrate drugs.

Beyond being a substrate of BCRP, MTX is also a substrate of multidrug resistance-associated proteins (MRPs), such as MRP2 [27] and several solute carrier (SLC) transporters, such as reduced folate transporter 1, proton-coupled folate transporter and organic anion-transporting polypeptide 1A2 [15,27]. Meanwhile, several studies have demonstrated that natural polyphenols (e.g., quercetin, epigallocatechin-3-gallate, kaempferol and resveratrol) and their metabolites (e.g., quercetin glucuronides and resveratrol glucuronides) are modulators of MRP2 and SLC transporters [28,29,30,31]. Furthermore, a previous study reported that PCRRM inhibited MRP2 and resulted in an intracellular accumulation of carbamazepine-10,11-epoxide [25], a MRP2 substrate [32]. Therefore, we speculate that the altered pharmacokinetics of MTX by PCRR coadministration might also involve MRP2, SLC transporters and others.

In rats, if PCRR was taken prior to or with MTX, both the BCRP in the intestine and kidney would be modulated and might lead to reduced absorption and the excretion of MTX. However, if PCRR was ingested a few hours later than MTX, the influence on the absorption process of MTX would be negligible, and the inhibition effect of PCRRM on BCRP in the kidney would be dominant, resulting in a more pronounced effect on increasing the systemic exposure of MTX than taking PCRR prior to or with MTX. Therefore, the timing of PCRR coadministration was very crucial to the influence on MTX pharmacokinetics. Taking PCRR a few hours later than MTX might enhance the toxicity of MTX and might even lead to life-threatening consequences. Although the dose selection of PCRR in rats was based on the daily dosage of PCRR in humans, direct translation of the modulation from rats to humans remains unknown and is the major limitation of this study.

We strongly suggest that the concurrent use of PCRR with MTX or other critical BCRP substrate drugs, such as carbamazepine and warfarin, should be avoided. In conclusion, PCRR activated BCRP, whereas PCRRM inhibited BCRP, and the coadministration of PCRR markedly reduced both the absorption and excretion of MTX in rats. In clinical practice, the concurrent use of PCRR with critical BCRP substrate drugs should be avoided.

## 4. Materials and Methods

### 4.1. Chemicals and Reagents

PCRR (the voucher specimen number of CMU-P-1905-10-II), deposited in China Medical University, was purchased from a drug store (Taichung, Taiwan) and assessed by Dr. Yu-Chi Hou. MTX (25 mg/mL) was supplied by Wyeth Pharma Gmbh (Wolfrats hausen, Germany). Mitoxantrone (MXR) and Ko143 were obtained from Enzo Life Sciences, Inc. (Farmingdale, NY, USA).

### 4.2. Animals

Male Sprague Dawley rats were obtained from BioLASCO Taiwan Co., Ltd. (Yi-Lan, Taiwan) and housed in a 12 h light–dark cycle, constant temperature environment at the Animal Center of China Medical University (Taichung, Taiwan) prior to the study. The animal study, including animal size selection, followed the guidelines outlined in “The Guidebook for the Care and Use of Laboratory Animals” published by the Chinese-Taipei Society for the Laboratory Animal Science (Taipei, Taiwan) and the pharmacokinetic guidelines of preclinical animal studies [33]. The dose selection of PCRR in rats was based on the daily dosage of PCRR in the 4th edition of the *Taiwan Herbal Pharmacopeia* [6] and the USFDA 2005 guideline [34]. This protocol was approved by the Institutional Animal Care and Use Committee (IACUC), China Medical University (Taichung, Taiwan) (protocol code: CMUIACUC-2023-289 and date of approval: 24 March 2023).

### 4.3. Preparation and Characterization of PCRR Decoction and PCRRM

The preparation and characterization of PCRR and PCRRM followed a previous study [25,35]. Briefly, the crude PCRR (250 g) was heated with an adequate amount of water and concentrated to achieve a final concentration of 0.5 g/mL PCRR decoction. Then, 20 µL of the PCRR sample was subject to HPLC analysis. On the other hand, rats (320–355 g) were orally administered the PCRR decoction (2.0 g/kg) and then blood was collected at 30 min after dosing. After centrifugation, the serum was vortexed with a 4-fold volume of methanol. Then, the supernatant was concentrated in a rotatory evaporator under vacuum to dryness. To the residue, an appropriate volume of water was added to afford a solution with 10-fold serum level. Then, 20 µL of the PCRRM sample was subject to HPLC analysis.

### 4.4. PCRR-MTX Pharmacokinetic Interaction in Rats

A parallel study was performed and rats (310–470 g) were randomly divided into three groups. Before the experiments, all rats were fasted overnight but drinking water was allowed ad libitum. Food was supplied 3 h after dosing. For group 1, rats were given MTX (5.0 mg/kg) alone, and for groups 2 and 3, rats were given MTX (5.0 mg/kg) orally with PCRR decoction (1.0 and 2.0 g/kg). Blood samples (0.5 mL) were collected at specific times after the oral intake of MTX. The remaining procedures were conducted in accordance with those described in a previous study [18].

### 4.5. Cell Culture Condition and Cell Viability Assay

A BCRP-transfected Madin–Darby canine kidney type II (MDCKII-BCRP) cell line was obtained from Prof. Dr. Piet Borst (Netherlands CancerInstitute, Amsterdam, The Netherlands). MDCKII-BCRP cells were grown in DMEM medium containing 10% of Hyclone^TM^ fetal bovine serum (Cytiva, Logan, UT, USA) and 1% of penicillin-streptomycin-glutamine (Invitrogen, Grand Island, NY, USA) at 37 °C in a humidified incubator with 5% CO_2_. In addition, the effects of the tested agents on cytotoxicity were performed by using an MTT (3-[4,5-dimethylthiazol-2-yl]-2,5 diphenyl tetrazolium bromide) assay and the procedures followed were those of a a previous study [18].

### 4.6. Function Assay of BCRP After Treatments with PCRR and PCRRM

MXR was utilized as a BCRP fluorescent substrate for investigating the function of BCRP [18]. Briefly, MDCKII-BCRP cells were incubated with a PCRR decoction and Ko143 (0.25 M, a positive BCRP inhibitor). Then, the MXR fluorescence was detected using an excitation wavelength of 607 nm and an emission wavelength of 684 nm. On the other hand, MDCKII-BCRP cells were pre-incubated with PCRRM and Ko143. Then, MXR was co-incubated and the fluorescence of MXR was determined by a FACScan flow cytometer.

### 4.7. Data Analysis

A noncompartment model with the assistance of Phoenix WinNonlin (version 8.4, Pharsight Corp., Cary, NC, USA) was utilized to determine the pharmacokinetic parameters. The statistical software SPSS (version 19) was used to analyze the variations in pharmacokinetic parameters among the treatments in rats via one-way ANOVA with a Scheffe test, while an unpaired Student’s *t*-test was employed for cell studies with significance set at *p* < 0.05.

## Figures and Tables

**Table 1:** Pharmacokinetic parameters of MTX (5.0 mg/kg) after dosing MTX alone and coadministrations with PCRR (1.0 g/kg and 2.0 g/kg) in rats.

	Treatments	MTX Alone	MTX + PCRR (1.0 g/kg) (*n* = 6)	MTX + PCRR (2.0 g/kg) (*n* = 5)
Parameters				
T_max_		25.0 ± 3.2	25.0 ± 3.2	18.0 ± 3.0
C_max_		0.35 ± 0.05 ^a^	0.23 ± 0.01 ^ab^	0.19 ± 0.02 ^b^ (−46%)
AUC_0~2880_		143.2 ± 21.2 ^ab^	106.8 ± 7.9 ^a^	181.8 ± 17.2 ^b^
AUC_0~240_		51.4 ± 6.6 ^a^	35.4 ± 1.5 ^b^ (−31%)	21.8 ± 1.2 ^b^ (−58%)
AUC_240~2880_		91.8 ± 17.7 ^a^	71.4 ± 7.6 ^a^	160.1 ± 17.4 ^b^ (+39%)
MRT_0~2880_		690.9 ± 79.7 ^a^	681.4 ± 79.4 ^a^	1134.3 ± 56.5 ^b^ (+74%)

Values are means ± S.E. Means in a row without a common superscript differ (a,b), *p* < 0.05. T_max_ (min): time to reach peak serum level. C_max_ (μmol mL^−1^): peak serum level. AUC_0-240_ (μmol min mL^−1^): area under the serum level–time curve from 0 to 240 min. AUC_240-2880_ (μmol min mL^−1^): area under the serum level–time curve from 240 to 2880 min. AUC_0-2880_ (μmol min mL^−1^): area under the serum level–time curve from 0 to 2880 min. MRT_0-2880_ (min): mean residence time from 0 to 2880 min.

## Data Availability

The raw data presented in this study will be shared on request from the corresponding author.

## Supplementary Materials

The following supporting information can be downloaded at: https://www.mdpi.com/article/10.3390/ph18111636/s1, Figure S1: HPLC chromatograms of resveratrol (RV), emodin (EM) and propyl paraben (PP, internal standard) in rat serum after PCRR ingestion; (A) Blank serum spiked with PP; (B) Serum sample after hydrolysis with sulfatase/glucuronides.

## Abbreviations

AUC_0-t_	Area under the serum level–time curve
BCRP	Breast cancer resistance protein
C_max_	Peak serum level
MDCKII-BCRP MRT	MDCKII transfected cells with overexpression of BCRP Mean residence time
MTX	Methotrexate
MXR	Mitoxantrone
PCRR	Polygoni Cuspidati Rhizoma et Radix
PCRRM	The serum metabolites of PCRR
