# Supplementary material for: Potential Modulation of Polygoni Cuspidati Rhizoma et Radix on Breast Cancer Resistance Protein and Marked Alteration on Methotrexate Pharmacokinetics
Source: Pharmaceuticals (Basel). 2025 Oct 29;18(11):1636. doi: 10.3390/ph18111636 (PMC12655440; doi:10.3390/ph18111636)
Supplement: Supplementary file 1 [file pharmaceuticals-18-01636-s001.zip › pharmaceuticals-3862314-supplementary.pdf]

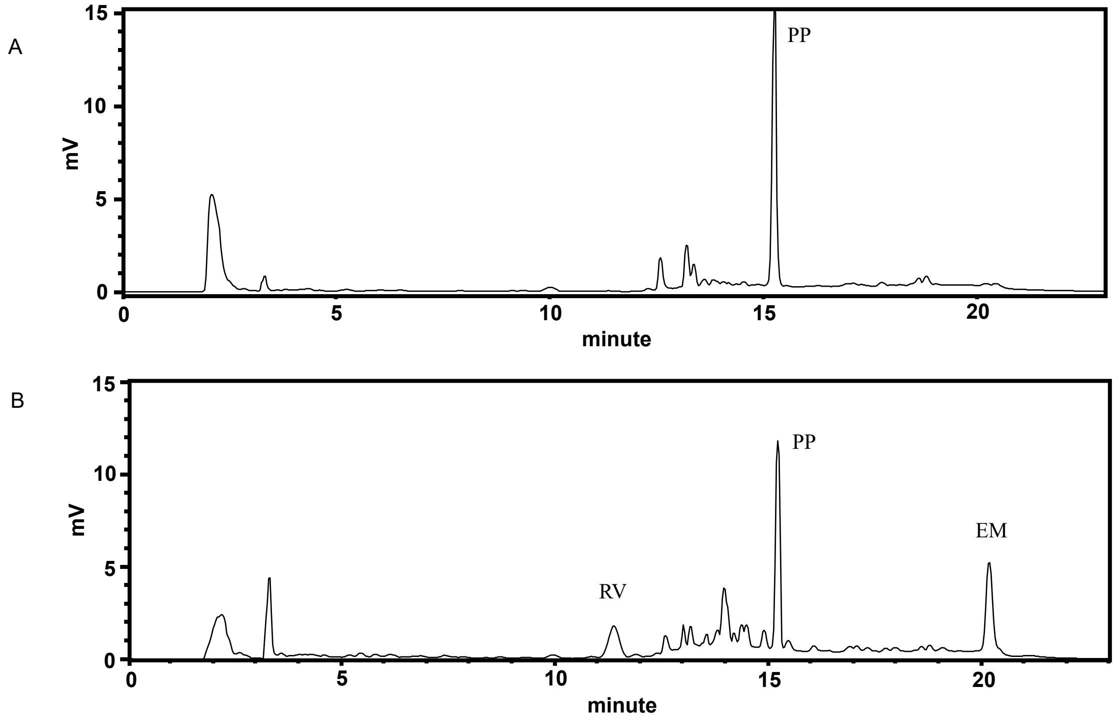

Fig. S1 HPLC chromatograms of resveratrol (RV), emodin (EM) and propyl paraben (PP, internal standard) in rat serum after PCRR ingestion.

(A) Blank serum spiked with PP

(B) Serum sample after hydrolysis with sulfatase/glucuronides
